# Physical and Psychological Factors Affecting Falls in Older Patients with Arthritis

**DOI:** 10.3390/ijerph17031098

**Published:** 2020-02-09

**Authors:** Mikyong Byun, Jiyeon Kim, Moonho Kim

**Affiliations:** 1 College of Nursing, Korea University, Anam-dong, Seongbuk-Gu, Seoul 02841, Korea; mulanbb@korea.ac.kr (M.B.); tortoi@korea.ac.kr (J.K.); 2 Department of Hematology and Oncology, Gangneung Asan Hospital, University of Ulsan College of Medicine, 38 Bangdong-gil, Sacheon-myeon, Gangneung-si, Gangwon-do 25440, Korea

**Keywords:** fall, arthritis, older adults, public health

## Abstract

As the population ages, falls are becoming one of the leading causes of morbidity and mortality. Joint disease (either osteoarthritis or rheumatoid arthritis) is a well-known predictor of falls, and these medical conditions increase in accordance with the aging population. This study aimed to describe individual, physical, and psychological characteristics between older adults with and without a fall history. Further, we aimed to identify statistically significant physical or psychological factors associated with falls by controlling individual variables. We analyzed data from the 2014 Survey of Living Conditions and Welfare Needs of Korean Older Adults. Adults aged 65 years or over with doctor-diagnosed joint disease were eligible. A total of 2707 women and 784 men (n = 3491) were enrolled. Of these, 1174 patients suffered a fall within a year (average number of falls = 2.4). We adopted individual variable-adjusted models and found that limited activities of daily living (odds ratio (OR) 1.4, 95% confidence interval (CI) 1.04–1.87), fear of falling (OR 7.18, 95% CI 4.26–12.09), and depression (OR 1.28, 95% CI 1.09–1.50) significantly increased fall risks on logistic regression analysis. Our findings suggest that physical and psychological factors, especially the fear of falling, need to be addressed to prevent falls in elderly patients with arthritis.

## 1. Introduction

In older adults, falls can cause severe injuries such as fractures and traumatic brain injuries. Underlying comorbidities can also result in falls, creating a vicious cycle. Hence, falls are now a serious public health problem, and about 30% of adults aged 65 and over fall each year [1,2]. Falls cause disabilities and mortality in older patients [3,4]. The rate of deaths from falls increased in the United States by an average of 3.0% per year during a period from 2007–2016 [5]. In South Korea, falls were the seventh specific cause of disability-adjusted life years (DALYs) according to the 2012 Korean Burden of Disease Study [6]. Moreover, fall-related hospitalizations led to substantial healthcare expenses and social burden. In the United States, the total costs from fatal and non-fatal falls in 2015 was $38 billion [7]. According to 2018 South Korea census data, there has been rapid growth in the aging population (the population aged 65 or older consists of 14.8 percent of the total population) [8]. As the population ages worldwide, direct and indirect costs due to falls are expected to increase in an exponential rather than linear way. 

Older age is known to be an important risk factor for falls [2,6]. Older patients are more vulnerable to falls, as most of the physical and psychological impairments are due to the inevitable aging process. Furthermore, the incidence of falls grows in patients with joint problems such as osteoarthritis (OA) and rheumatoid arthritis (RA). Since arthritis causes pain and impaired mobility in the joints, it adversely affects body balance and postural stability. A large amount of research has presumed that patients with arthritis are susceptible to falls. For example, OA is a major predisposing factor for increased falls [9,10,11,12], and RA, the second most common type of arthritis after OA, is also reported to increase the risk of falls [13,14,15]. 

Several predictive factors were found to influence falls in either OA or RA studies. Of these, the most consistent variable has been an advanced age [2,9]. In the literature review, most falls and joint disease studies focused only on OA or RA respectively for their study population [2,9]. Few of them encompassed both OA and RA together, especially in the elderly population [16]. In addition, the proposed fall risks were not always well organized and were sometimes conflicting. 

This study aims to describe individual, physical, and psychological characteristics between older joint disease patients with and without a fall history. In addition, we tried to identify statistically significant physical or psychological factors associated with falls.

## 2. Materials and Methods 

### 2.1. Data Sources

Public data were gathered from the Health and Welfare Data Portal in Korea under the approval of the National Statistical Office. The data originated from the 2014 National Survey of Living Conditions and Welfare Needs of Korean Older Adults [17]. The database was made up of a stratified random sample of approximately ten thousand people who were living in general housing facilities and was designed to be representative of the Korean elderly population. The Ministry of Health and Welfare conducted this survey from June to September in 2014. Trained investigators visited and interviewed adults aged 65 and over regarding their living status and welfare needs in residential areas including 16 cities and provinces. 

### 2.2. Patient Selection and Study Design

A total of 3491 patients with arthritis were identifiable from the original data of 10,451 participants. Patients with arthritis were defined as those who responded affirmatively to the questions, “have you been diagnosed with osteoarthritis or rheumatoid arthritis by a doctor?” and “have you have arthritis for more than three months?”. 

Using a descriptive and correlational study design, we first evaluated predictors of falls from three main categories (individual, physical, and psychological). We then adopted individual variable-adjusted models to find major physical and psychological factors that increased the fall risk on logistic regression analysis.

### 2.3. Ethical Considerations

Our Institutional Review Board (IRB) determined that this project was exempt from IRB review because the research used existing national data and the information could not be linked to individual subjects (IRB No. 2019-11-018). Informed consent was not required as data were de-identified and collected retrospectively.

### 2.4. Measurements

#### 2.4.1. Fall History

Fall history was evaluated using a single question: “have you experienced a fall (stumble, slip, or fall down) within a year?”. The respondents could answer that they “experienced a fall” or “did not experience a fall” [17].

#### 2.4.2. Individual Variables

The individual characteristics were classified into four subcategories: demographic, socio-economic, health status, and health-related behavior. First, demographic variables included age, sex, marital status (living with spouse or living without spouse), and living status (alone, living with spouse, or living with children). Second, socio-economic variables were the subject’s education level (0–6 years, 7–9 years, 10–12 years, or ≥13 years), employment (employed or not employed), and quartiles of household income (Q1 (lowest), Q2, Q3, or Q4 (highest)). Third, heath status variables involved chronic diseases (hypertension, diabetes, or dementia), body mass index (BMI), and number of medications (0, 1, 2, or ≥3). Finally, health-related behaviors consisted of exercise (none, <150 min a week, or ≥150 min a week), smoking (past/never or current), and drinking (none, ≤1 standard drink/day, or >1 standard drink/day).

The presence of chronic disease was investigated using the question, “have you currently been suffering from hypertension, diabetes or dementia for more than three months?” and “have you been diagnosed by a doctor?”. Participants who answered “yes” to both questions were classified as having a chronic disease. The number of medications was based on the response to the question, “how many physician-prescribed drugs have you been taking for the past three months or more?”. Exercise was considered to be within the recommended levels in those who exercised for more than 150 min a week, according to the World Health Organization (WHO) criteria [18]. Exercise levels were classified as within the recommended level, below the recommended level, and none. Responses of “no” to “do you usually exercise?” were classified as “none”. Drinking status was defined based on the National Institute on Alcohol Abuse and Alcoholism criteria [19]. In older people aged 65 and over, drinking one standard drink (A 350 mL glass of beer) of alcohol per day is considered to be appropriate intake in Korea; intake of more than one standard drink of alcohol per day is considered severe. Those who do not drink alcohol at all are classified as “none”.

#### 2.4.3. Physical Variables

Physical characteristics included visual impairment, hearing impairment, limited activities of daily living (ADL), and limited instrumental activities of daily living (IADL). 

Sensory impairments were classified as either visual or auditory. Visual impairment was classified as “not impaired” in those who responded that they felt comfortable not wearing glasses, lenses, or using magnifying glasses during daily activities. Those responding “uncomfortable” and “very uncomfortable” were classified as “impaired”. Hearing impairment was classified as “not impaired” in individuals who did not require hearing aids in daily life without any discomfort. Those who responded “uncomfortable” and “very uncomfortable” were classified as “impaired”. 

The evaluation of ADL was based on the Korean Activity Daily Living scale [20]. This consists of seven questions: “dressing”, “face washing, brushing teeth, and shampooing”, “bathing”, “eating food”, “getting up and walking across the room”, “toilet use”, and “bowel and bladder control”. It also includes a three-point scale (total independence/partial dependence/total dependence). Total independence was recorded as “no limitation”, and partial and complete dependence were regarded as “limitation”. Subjects who were identified as having restrictions of ADL in more than one item were classified as having a “limitation of ADL”. 

Limitations in IADL were determined using the Korean Instrumental Activity of Daily Living scale [20]. This consists of ten questions: “grooming”, “doing housework”, “preparing meals”, “washing clothes”, “picking up a set amount of medicine on time”, “managing money”, “going out to a nearby place”, “making purchasing decisions, paying money, and receiving change”, “making and receiving phone calls”, and “using transportation”. It also includes three- and four-point scales. The three-point scale (Items 1–7) includes independence/partial dependence/total dependence, while the four-point scale (Items 8–10) includes independence/little dependence/much dependence/cannot be done at all. Total independence was classified under “no limitation”, and partial, complete, little, much dependence, and cannot be done at all were recorded as “limitation”. Subjects who were classified as having restrictions in IADL in more than one item were regarded as having a “limitation of IADL”. 

Nutrition was determined using the “Determine Your Nutritional Health” questionnaire of the Nutrition Screening Initiative [21]. This consists of ten questions with binary responses of “yes” or “no”. A “yes” response to each question is scored in the range of 1–4; a “no” response is scored 0 points. The total score of 10 items is classified as 0–2: good nutrition, 3–5: moderate nutritional risk, and ≥6: high nutritional risk. Good nutrition scores were considered to be “good nutrition”, and moderate and high nutritional risk were defined as “poor nutrition.”

#### 2.4.4. Psychological Variables

Psychological characteristics were primarily fear of falling (FOF) and depression. The FOF was investigated using the question, “are you usually afraid of falling?”. Participants were asked to choose one response on a three-point scale: not at all, sometimes, or mostly, with a higher score indicating a greater FOF. A “not at all” response was considered as “no FOF”, and “sometimes” and “mostly” were regarded as “have FOF” during data analysis. Depression was determined using the Korean version of the 15-item Geriatric Depression Scale K (SGDS-K), which was proposed by Sheik and Yesavage and was translated into Korean by Cho et al. [22]. On this scale, scores ranged from 0 to 15. A previous study from Korea suggested the optimal cut-off for SGDS-K scores during screening for major depressive disorders to be ≥8; in this study, scores of ≥8 and <8 were classified as “depressed” and “not depressed”, respectively.

### 2.5. Data Analysis

**Figure 1:**
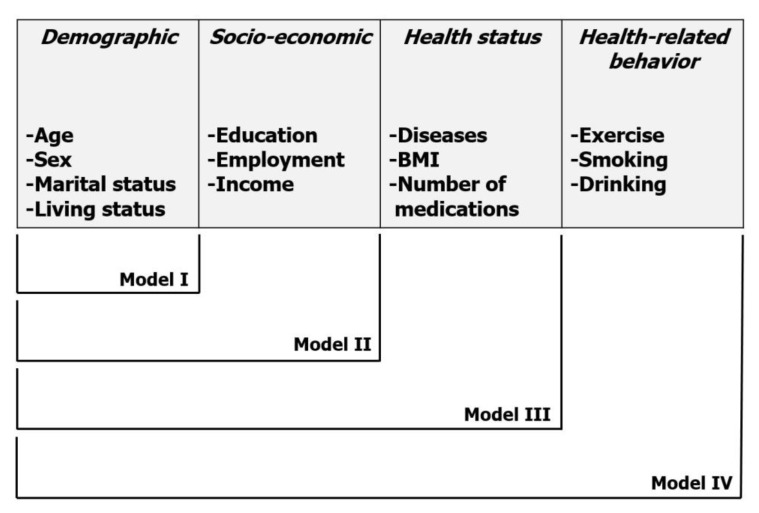
Suggested individual variable-adjusted models. The variables from individual categories, including demographic, socio-economic, health status, and health-related behavior, were combined in groups, and four models were established.

Descriptive statistics were performed; differences in the fall history by individual, physical, and psychological variables were compared using the χ2 or *t*-test. Each independent variable was included for univariate logistic regression analysis, from which significant variables were chosen for multivariate logistic regression analysis. In order to control the possible confounding effects of individual factors, we applied individual variable-adjusted models. The variables from the individual category, including demographic, socio-economic, health status, and health-related behavior subcategories, were combined in groups and entered into the logistic regression models (Model I, demographic only; Model II, demographic and socio-economic; Model III, demographic, socio-economic, and health status; Model IV, demographic, socio-economic, health status, and health-related behavior) (Figure 1). Odds ratios (ORs) and corresponding 95% confidence intervals (CIs) were also calculated through analysis. The level of statistical significance was set at less than 0.05. The data were analyzed using the IBM SPSS statistical software Version 22.0 (IBM, Armonk, NY, USA) package.

## 3. Results

### 3.1. Incidence of Falls

A total of 1174 patients with arthritis (33.6%) experienced a fall within a year, and the average number of falls was 2.4 events per year.

### 3.2. Differences in Individual Characteristics of Arthritis Patients with and without a Fall History

Differences in fall history according to individual characteristics are summarized in Table 1. In terms of demographic, socio-economic, health status, and health-related behavior characteristics, all factors (except hypertension, diabetes, and BMI) were statistically significant between the fall and non-fall groups in our study.

### 3.3. Differences in Physical and Psychological Characteristics of Arthritis Patients with and without a Fall History

There were significant differences between the two groups in terms of physical characteristics such as visual, hearing, ADL, IADL, and nutrition status (Table 2). Older adults with visual or hearing impairments were more vulnerable to falls, and the incidence of falls was higher in those with ADL or IADL limitations. 

There were also significant differences between the two groups in view of psychological factors such as FOF and depression (Table 2). Almost all patients with arthritis (98.6%) had a FOF, and more than half (53.2%) had depressive episodes in the fall subgroup.

### 3.4. Multivariate Logistic Regression Analysis of Physical and Psychological Factors Using Individual Variable-Adjusted Models

To evaluate the physical or psychological factors affecting risks for fall, we introduced four individual variable-adjusted models, as mentioned above. In Model IV, our study revealed that the fall risks among older adults with arthritis were significantly associated with ADL limitation (OR: 1.40, 95% CI 1.04–1.87), FOF (OR: 7.18, 95% CI 4.26–12.09), and depression (OR 1.28, 95% CI 1.09–1.50) on multivariate analysis (Table 3). Of note, poor nutrition (OR 1.18, 95% CI 1.00–1.40) was found to be associated with the risk of falling in Model I only.

## 4. Discussion

In this study, we sought to identify physical or psychological factors associated with a risk of falls in older patients with joint disease by controlling individual factors. We identified statistically significant predictors of falls (i.e., ADL limitation, FOF, and depression), and we believe that these factors need to be addressed to prevent falls in elderly patients with arthritis.

In a recent report using a Korean nationwide survey (2010–2012), the prevalence of degenerative OA in males and females was 9.3% and 28.5%, respectively [23]. RA is an autoimmune form of joint disease and is the second most common cause of arthritis. The prevalence of RA in South Korea estimates was 0.27% in 2008 [24]. Interestingly, contrary to previous results regarding the relevance of falls and arthritis, there have been different opinions from some experts [25,26]. Some OA studies reported no association between knee pain and a fall, and they even suggested that pain was protective against falls [27,28,29,30]. Similarly, the severity of RA was not found to be linked to falls in several studies [13,15,31,32,33]. Thus, we may need to keep in mind that differing arguments exist regarding the correlation between falls and joint conditions. Obviously, OA and RA are different disease entities with distinctive pathophysiologies. When it comes to the limited range of motion and muscle weakness in an elderly person, arthritis itself (both OA and RA) can play an important role in falls. To the best of our knowledge, almost all studies on this topic focused on only one specific disease entity (either OA or RA). Few studies covered both OA and RA patients together with respect to falls [16]. Quach LT et al. examined the association between different types of arthritis (22% OA, 4.8% RA, 2.3% both OA and RA, and 7.9% with other arthritis types) and falls and investigated whether depression symptoms moderated these relationships. They showed that there was no significant interaction between the types of arthritis and depression symptoms. Many potential risk factors for falls were proposed in OA or RA studies. Of these, the most consistent factor has been old age, regardless of two disease conditions. In our study, we included 3491 older adults diagnosed with arthritis (either OA or RA were eligible) and analyzed variables according to individual, physical, and psychological categories in this context. 

Regarding individual characteristics including demographic, socio-economic, health status, and health-related behavior, all variables (except hypertension, diabetes, and BMI) were statistically significant between the fall group and the non-fall group. In physical variables, we found that visual impairment, hearing impairment, limitation of ADL, limitation of IADL, and poor nutrition were significant. Lastly, depression and FOF were independent psychological risk factors for falls. We next tried to demonstrate relevant physical or psychological factors associated with falls by controlling individual variables. To set up risk-adjusted models, individual factors were divided into four subcategories: demographics (such as age, sex, and marital status), socio-economic status (such as education and household income), health status (such as disease and number of medications), and health-related behavior (such as exercise and smoking). As mentioned above, instead of combining these subcategories at the same time, we added subgroups one by one to find out which combinations (or models) would be helpful in controlling possible individual confounders. We also identified factors that were coherent throughout the four models. As a result, we were able to demonstrate that three factors (limitation of ADL, FOF, and depression) were significantly associated with the risk of falls, and FOF seemed to be the most powerful among them. 

FOF has been recognized as one of the important risk factors for falls in older adults [34,35,36]. Further, elderly arthritic patients who had experienced a fall were more likely to have FOF than who had not. Of course, FOF can be a result of a fall. Simply managing the fear itself may not be effective in fall prevention; a recent systematic review and meta-analysis emphasized that multifactorial interventions (mostly exercise prescriptions) may reduce the rate of falls in older people [37]. Previous fall experiences make older arthritis patients feel more intimidated and restricted in their usual activities [36,38]. The resulting social isolation and avoidance of activities by FOF might eventually lead to depressive episodes. A study reported that 37.5% of older adults with a history of falls with a moderate or severe FOF had a concurrent depressive mood disorder [39]. In contrast, antidepressant medications and sedatives can increase the risk of falls. Depression and falls have a complex and bidirectional relationship [40]. Likewise, in our study, an increased risk of falls was statistically associated with both FOF and depression. 

In regard to patients’ physical variables like ADL and IADL, which reflects the functional capacities of the elderly, we found that there was a relationship between fall experience and a limitation of ADL, which was consistent with prior studies [41,42].

There were some limitations to this study. First, there may be a recall bias, as the source of this study’s data was interview results. Second, this data analysis had a cross-sectional design, which may not always find the causality. In other words, the cause and effect relationship between falls and arthritis could be unknown, which was an inherent limitation of this design. Third, to simplify our hypothesis, we did not take into account environmental variables (such as residential area, housing type, and so on), which were considered in other FOF studies [43,44]. 

Despite these limitations, our fall study was the first nationwide data analysis to incorporate both OA and RA elderly patients. We also investigated physical and psychological risk factors of falls using multi-step variable-adjusted models.

## 5. Conclusions

Falls are a worldwide public health issue among the elderly population. Our findings suggested that physical and psychological factors, especially FOF, limitation of ADL, and depression, need to be addressed to prevent falls in elderly patients with arthritis. In addition, our results necessitate further validation by well-designed prospective clinical trials with a larger cohort. All authors have read and agreed to the published version of the manuscript.

## Figures and Tables

**Table 1:** Differences in individual characteristics between those with and without a history of falls (n = 3491).

Variables		Classification	Without Fall	With Fall	*t* or χ^2^	*p*
			(n = 2317)	(n = 1174)		
			n (%) or	n (%) or		
			M ± SD *	M ± SD *		
Demographic	Age		74.5 ± 6.2	75.5 ± 6.7	19.89	<0.001
	Sex	Male	584 (25.2)	200 (17.0)	29.99	<0.001
		Female	1733 (74.8)	974 (83.0)		
	Marital status	Living with spouse	1249 (53.9)	508 (43.3)	34.94	<0.001
		Living without spouse	1068 (46.1)	665 (56.7)		
	Living status	Alone	723 (31.2)	420 (35.8)	18.98	<0.001
		Living with spouse	974 (42.0)	404 (34.4)		
		Living with children	620 (26.8)	350 (29.8)		
Socio-economic	Education	0–6 years	1750 (75.5)	957 (81.5)	18.22	<0.001
		7–9 years	275 (11.9)	117 (10.0)		
		10–12 years	223 (9.6)	79 (6.8)		
		≥13 years	69 (3.0)	20 (1.7)		
	Employment	Employed	671 (29.0)	254 (21.6)	21.56	<0.001
		Not employed	1646 (71.0)	920 (78.4)		
	Quantiles of household income	Q1 (lowest)	275 (11.8)	231 (19.7)	45.61	<0.001
		Q2	996 (43.0)	511 (43.5)		
		Q3	996 (43.0)	410 (34.9)		
		Q4 (highest)	50 (2.2)	22 (1.9)		
Health status	Disease	Hypertension	1397 (60.3)	746 (63.6)	3.58	0.058
		Diabetes	532 (22.9)	292 (24.9)	1.63	0.202
		Dementia	63 (2.7)	57 (4.8)	10.44	<0.001
	BMI **		23.9 ± 3.3	23.7 ± 3.5	1.34	0.246
	Number of medications	0	252 (10.9)	102 (8.7)	14.36	<0.01
		1	197 (8.5)	67 (5.7)		
		2	167 (7.2)	81 (6.9)		
		≥3	1701 (73.4)	924 (78.7)		
Health-related Behavior	Exercise	None	1166 (50.3)	606 (51.7)	21.36	<0.001
		<150 min a week	333 (14.4)	227 (19.3)		
		≥150 min a week	818 (35.3)	341 (29.0)		
	Smoking	Past/never	2090 (90.2)	1091 (93.0)	7.39	<0.01
		Current	227 (9.8)	83 (7.0)		
	Drinking	None	1884 (81.3)	1018 (86.7)	22.31	<0.001
		≤1 standard drink/day	232 (10.0)	103 (8.8)		
		>1 standard drink/day	201 (8.7)	53 (4.5)		

* M ± SD, mean ± standard deviation. ** BMI, body mass index. Note: the total number of participants is slightly different for the non-response items.

**Table 2:** Differences in physical and psychological characteristics between those with and without a history of falls (n = 3491).

Physical & Psychological Variables		Classification	Without Fall	With Fall	χ^2^	*p*
			(n = 2317)	(n = 1174)		
			n (%)	n (%)		
Physical	Visual impairment	No	1294 (55.9)	569 (48.5)	16.45	<0.001
		Yes	1023 (44.2)	605 (51.5)		
	Hearing impairment	No	1707 (73.8)	789 (67.2)	16.27	<0.001
		Yes	610 (26.2)	385 (32.8)		
	ADL * limitation	No	2151 (92.8)	1015 (86.5)	37.34	<0.001
		Yes	166 (7.2)	159 (13.5)		
	IADL ** limitation	No	1822 (78.6)	799 (68.1)	46.24	<0.001
		Yes	495 (21.4)	375 (31.9)		
	Nutrition	Good	1027 (44.3)	402 (34.3)	32.45	<0.001
		Poor	1290 (55.7)	772 (65.7)		
Psychological	Fear of falling	No	271 (11.7)	16 (1.4)	107.30	<0.001
		Yes	2046 (88.3)	1158 (98.6)		
	Depression	No	1402 (60.5)	550 (46.9)	57.96	<0.001
		Yes	915 (39.5)	624 (53.2)		

* ADL, activities of daily living; ** IADL, instrumental activities of daily living.

**Table 3:** Multivariable logistic regression analysis of factors associated with falls in older adults with arthritis.

Variables	Model I	Model II	Model III	Model IV
	OR (95% CI)	OR (95% CI)	OR (95% CI)	OR (95% CI)
Visual impairment	1.09 (0.93–1.24)	1.06 (0.91–1.23)	1.06 (0.91–1.23)	1.07 (0.92–1.25)
Hearing impairment	1.17 (0.98–1.39)	1.17 (0.98–1.39)	1.18 (0.99–1.40)	1.18 (0.99–1.40)
ADL limitation	1.36 (1.022–1.82) *	1.40 (1.01–1.87) *	1.37 (1.03–1.84) *	1.40 (1.04–1.87) *
IADL limitation	1.18 (0.96–1.44)	1.17 (0.96–1.43)	1.17 (0.96–1.43)	1.17 (0.96–1.44)
Poor nutrition	1.18 (1.00–1.40) *	1.15 (0.97–1.36)	1.14 (0.96–1.35)	1.14 (0.96–1.35)
Fear of falling	7.35 (4.38–12.34) ***	7.40 (4.40–12.46) ***	7.38 (4.38–12.42) ***	7.18 (4.26–12.09) ***
Depression	1.40 (1.20–1.63) ***	1.28 (1.09–1.51) **	1.28 (1.09–1.50) **	1.28 (1.09–1.50) **

Model I: adjusted for demographic (age, sex, marital and living status) characteristics only. Model II: adjusted for demographic and socio-economic (education, employment, household income) characteristics. Model III: adjusted for demographic, socio-economic, and health status (disease, BMI, number of medications) characteristics. Model IV: adjusted for demographic, socio-economic, health status, and health-related behavior (exercise, smoking, drinking) characteristics. * *p* < 0.05, ** *p* < 0.01, *** *p* < 0.001.
